# Safe distances between groundwater-based water wells and pit latrines at different hydrogeological conditions in the Ganges Atrai floodplains of Bangladesh

**DOI:** 10.1186/s41043-016-0063-z

**Published:** 2016-08-19

**Authors:** M. Sirajul Islam, Zahid Hayat Mahmud, M. Shafiqul Islam, Ganesh Chandra Saha, Anwar Zahid, AHM Zulfiquar Ali, M. Qumrul Hassan, Khairul Islam, Hasin Jahan, Yakub Hossain, M. Masud Hasan, Sandy Cairncross, Richard Carter, Stephen P. Luby, Alejandro Cravioto, Hubert Ph. Endtz, Shah M. Faruque, John D. Clemens

**Affiliations:** 1 International Centre for Diarrhoeal Disease Research, Bangladesh, Mohakhali, Dhaka, 1212 Bangladesh; 2 Dhaka University of Engineering and Technology, Gazipur, Bangladesh; 3 Hydrogeology and Environmental Geology Section, Department of Geology, University of Dhaka, Dhaka, 1000 Bangladesh; 4 Directorate of Groundwater Hydrology, Bangladesh Water Development Board, Dhaka, 1205 Bangladesh; 5 Department of Soil, Water and Environment, University of Dhaka, Dhaka, 1000 Bangladesh; 6 WaterAid Bangladesh, Banani, Dhaka, 1213 Bangladesh; 7 Village Education Resource Center, Savar, Dhaka, 1340 Bangladesh; 8 London School of Hygiene and Tropical Medicine, London, UK; 9 WaterAid, London, UK; 10 Department of Medical Microbiology and Infectious Diseases, Erasmus Medical Centre, Rotterdam, The Netherlands; 11 Environmental Microbiology Laboratory, Laboratory Sciences and Services Division, icddr,b, GPO Box-128, Dhaka, 1000 Bangladesh

**Keywords:** Bacterial contamination, Hydrogeological condition, Pit latrine, Safe distance, Tubewells

## Abstract

**Background:**

Groundwater drawn from shallow tubewells in Bangladesh is often polluted by nearby pit latrines, which are commonly used toilets in rural and sub-urban areas of the country.

**Methods:**

To determine the minimum safe distance of a tubewell from a pit latrine in different hydrogeological conditions of Bangladesh, 20 monitoring wells were installed at three study sites (Manda, Mohanpur and Bagmara) with the vertical and horizontal distances ranging from 18–47 to 2–15 m, respectively. Water samples were collected three times in three seasons and tested for faecal coliforms (FC) and faecal streptococci (FS) as indicators of contamination. Soil samples were analysed for texture, bulk density and hydraulic conductivity following standard procedures. Sediment samples were collected to prepare lithological logs.

**Results:**

When the shallow aquifers at one of the three sites (Mohanpur) were overlained by 18–23-m-thick aquitards, the groundwater of the monitoring wells was found contaminated with a lateral and vertical distances of 2 and 31 m, respectively. However, where the aquitard was only 9 m thick, contamination was found up to lateral and vertical distances of 4.5 and 40.5 m, respectively. The soil textures of all the sites were mainly composed of loam and sandy loam. The hydraulic conductivities in the first aquifer at Manda, Mohanpur and Bagmara were 5.2–7.3, 8.2 and 1.4–15.7 m/h, respectively.

**Conclusions:**

The results showed that the safe distance from the tubewell to the pit latrine varied from site to site depending on the horizontal and vertical distances of the tubewell as well as hydrogeological conditions of a particular area.

## Background

Excreta-related diseases and deaths of children have been a major concern and guiding factor in national plans for public health in Bangladesh. Disposal of (under-5) children’s faeces into latrines is done by only a small minority of households in rural areas and slum dwellings. The problem is further complicated by very limited knowledge about the link between sanitation facilities, a safe environment, and illness [1, 2]. However, contamination of a well can also occur as a result of poor well design and/or construction [3].

Groundwater sources are often contaminated by pit latrine when the safe distance between a water point and pit latrine is not adequately maintained. Microbial contamination and water-borne diseases are caused by improper sanitation system in many developing countries including Bangladesh [4, 5]. Different studies mentioned that about 50 % of the water samples collected from shallow tubewells in Bangladesh were contaminated with human faecal organisms [6–8]. In Bangladesh, pit latrines are generally constructed close to tubewells, mainly due to space constraint, hygiene and convenience. Widespread use of pit latrines in rural and suburban areas makes them a major source of groundwater contamination. Effluent from pit latrines contains pathogenic bacteria, viruses, protozoa and helminths. The pathogens from the pit latrine may filtrate through the ground (unsaturated and saturated) and ultimately reach the groundwater [9, 10]. Infectious diseases like cholera, typhoid, dysentery and other diarrhoeal diseases are common in Bangladesh, killing more than 20,000 children annually [11].

The main contaminants from a pit latrine are the microorganisms present in the pit. Distance between a tubewell and a latrine and local geological and hydrogeological conditions are important factors for bacterial spread contaminating the tubewell [12–14]. However, these contributing factors have not been adequately studied in Bangladesh. When the organisms leach out into the soil, amongst other factors, the hydraulic conductivity of the soil (i.e. the volume of water that moves in a unit time under a unit hydraulic gradient through a unit area), determines how the organisms move to the saturated zone of groundwater. The hydraulic conductivity of the soil again depends on its particle size, but also on textural factors such as the horizontal layering formed by the annual deposition of silt. In this paper, the term soil refers to the upper most 3 m of the earth surface and the term sediment refers to underline material. The other important factors that influence transport of bacteria in aquifer systems are the physical transport processes of advection and hydrodynamic dispersion and microbe decay [15]. The transport of microbiological pathogens in groundwater is limited by die-off and attenuation (including filtration and adsorption). The processes of die-off and attenuation of bacteria occur in all groundwater aquifers [16]. In a sand and gravel aquifer, coliforms have been isolated 30 m from the source within 35 h of initial contamination [17]. Some pathogenic bacteria have been shown to persist in soil for up to 42 days [18]. Faecal bacteria are, therefore, frequently found at much greater distances and depths than predicted [10]. Therefore, in-depth field investigation is required to determine the conditions and environment of spread of bacteria in the subsurface.

Previous studies have been conducted in Bangladesh mainly by collecting water samples from the existing wells in the vicinity of pit latrines [6–8, 19, 20]. No study has been conducted installing and monitoring wells around pit latrines considering different hydrogeological conditions in order to monitor microbial movement for preparing guidelines to establish the safe distance of a tubewell from the nearest pit latrine. Therefore, the present study was carried out to determine a minimum safe distance between a tubewell and a pit latrine under different hydrogeological conditions in the Ganges Atrai flood plain areas of Bangladesh.

## Methods

### Study area

**Fig. 1:**
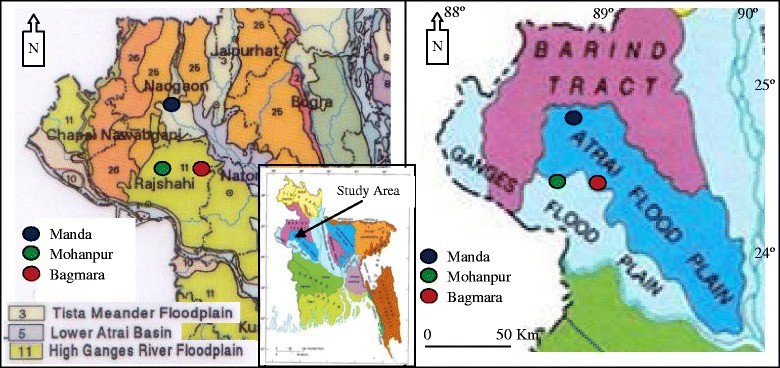
Agroecological regions and surface hydrogeological map showing location of study upazilas (sub-districts)

The study was conducted during the period from March to December 2008 in three upazilas (sub-districts) namely Manda of Naogaon district and Mohanpur and Bagmara of Rajshahi district of Bangladesh. The monsoon season (May to August) is included in the study period. Hydrogeologically, the study sites fall under the Ganges and Atrai flood plains bordered by the Barind Tract from the west, north and north-east [21] (Fig. 1). The soils of the study areas cover the agroecological regions [22] of the Tista Meander Floodplain, Lower Atrai Basin and High Ganges River Floodplain (Fig. 1). The tubewell water was free from arsenic and iron contamination. The depths of the latrines varied from 2 to 2.5 m. The water level in Manda, Mohanpur and Bagmara varied from 2–9.5, 4–14 and 1.5 to 10 m, respectively, during the study period.

### Establishment of monitoring wells

**Fig. 2:**
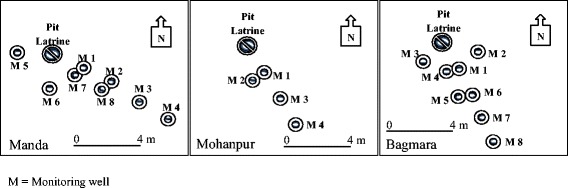
Distribution of monitoring wells from pit latrine at Manda, Mohanpur and Bagmara. *M* monitoring well

**Fig. 3:**
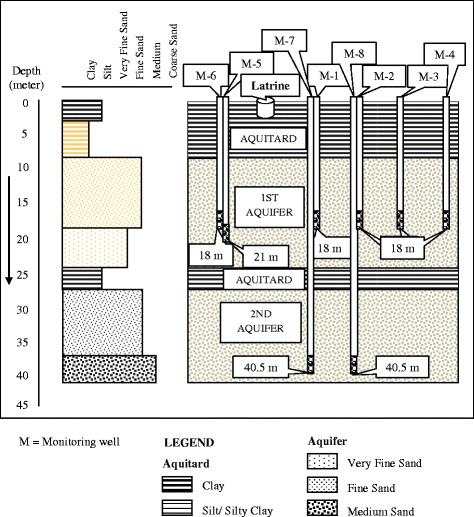
Types of sediments and depths of monitoring wells at Manda. *M* monitoring well

**Fig. 4:**
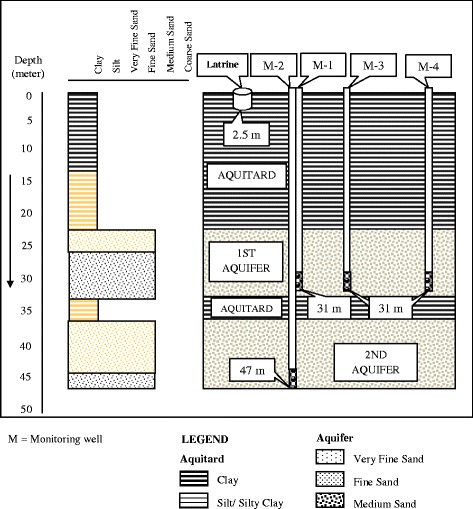
Types of sediments and depths of monitoring wells at Mohanpur. *M* monitoring well

**Fig. 5:**
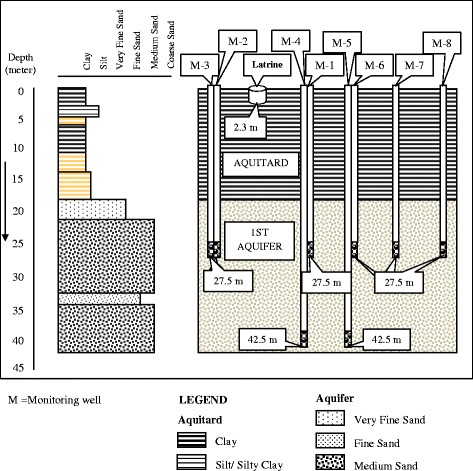
Types of sediments and depths of monitoring wells at Bagmara. *M* monitoring well

The nest of monitoring wells in Manda upazila was installed at Master Para in Kusumba union. The area is located on the bank of the river Atrai and the river flows towards the southeast. Wells were installed at Manda, Mohanpur and Bagmara to monitor the groundwater flow from a target latrine at each site. Monitoring wells were installed along the groundwater flow path from the latrine. Each latrine was used by five persons or more for at least 1 year. The flow path was predicted by the examination of the local disposition of surface water, hand-tube wells and pumping irrigation wells. The arrangement and spatial disposition of the monitoring wells are shown in plain view in Fig. 2 and in cross-section (to show depth) in Figs. 3, 4 and 5.

### Collection and analysis of samples

Soil samples were collected from three study upazilas by pit method until the water table was reached. Hydraulic conductivity rating of sediment was determined according to the method described by O’Neal [23]. Bulk density was determined by core sampling method, and bulk samples were used for particle size analysis. A metallic core of known volume was pressed or driven into the soil at the desired depth and thus an undisturbed soil sample was collected. Mass of the soil sample was found by weighing after oven drying the soil. The volume was calculated from the core dimension used for drawing the sample. Particle size analysis was determined by the hydrometer method [24]. Hydraulic conductivity was determined following the procedure described by Klute [25].

Sediment samples were collected during drilling of the wells and used to prepare the lithological logs in order to identify the sediment type and extension of the aquifers and aquitard. Manual hand percussion method was used by local drillers to drill the wells. Samples were collected from every 1.5-m depth. Representative samples were selected for sieve analysis of aquifer sediments to determine physical and hydraulic properties. The uniformity coefficient (Uc), i.e. D60/D10, of the sediment samples was calculated from grain-size analysis. The hydraulic conductivity of aquifer sediments was determined from the grain size distribution curve following Hazen’s method [26].

Water samples were collected from existing tubewells and from the established monitoring wells which were installed 1 week earlier following procedures described earlier [27, 28]. In brief, the tubewell mouths were first cleaned using tissue paper. The interior of the pump spout was sterilised using alcohol and a gas burner. The tubewell water was pumped out and allowed to flow for 2 min. Then, 500-ml water samples were aseptically collected in sterile Nalgene plastic bottles. All samples were transported directly to the Environmental Microbiology Laboratory of International Centre for Diarrhoeal Disease Research, Bangladesh (icddr,b) in an insulated box filled with cool packs (Johnny Plastic Ice, Pelton Shepherd, Stockton, CA, USA) and processed within 24 h. The monitoring wells were sampled three times to cover three seasons during the study period.

The FC and FS were counted following procedures described elsewhere [6, 28]. In brief, for FC and FS, 100-ml water samples were filtered through a 0.22-μm pore-size membrane filter (Millipore Corp., Bedford, MA, USA), and the filters were placed on membrane faecal coliforms (mFC) and KF-streptococcus agar plates. The mFC plates were incubated at 44 °C for 18 to 24 h. Then, the characteristic blue colonies were counted as FC and expressed as colony-forming unit (CFU) per 100 ml. The KF-streptococcus agar plates were incubated at 37 °C for 48 h, and the characteristic light and dark red colonies were counted as FS.

## Results

### Manda study site

The monitoring well logs in Manda showed that the upper or first aquifer is extended up to the depth of 25 m and is dominated by brown and grey, fine-to-very-fine sand and overlain by a 9-m-thick silty clay layer (Fig. 3). The lower or second aquifer was encountered below 27–40.5 m from the surface, consisting of grey and fine-to-medium sand. A 2-m-thick grey silty clay layer separated the first and the second aquifers.

The soil texture of Manda was composed of mainly loam and sandy loam (Table 1). The bulk density of soil was between 1.15 and 1.58 gm/cm^3^, respectively. The saturated hydraulic conductivity of sediment was measured from 2.30 to 518 mm/h. The calculated Uc of the aquifer sediments was 1.23–1.64 for the first aquifer and 1.96–3.16 for the second aquifer (Table 2). Hydraulic conductivities were 5.2–7.3 and 3.6–11.7 m/day for the first and the second aquifer sediments, respectively.

**Table 1:** Physical properties of soil samples at Manda, Mohanpur and Bagmara

Location	Depth (m)	Texture	Bulk density (gm/cm^3^)	Saturated hydraulic conductivity (mm/h)
Manda	0	Sandy loam	1.56	15.60
	0.15	Loam	1.58	2.30
	0.30	Sandy loam	1.47	5.20
	0.60	Loam	1.41	5.50
	1.25	Sandy loam	1.47	3.50
	1.85	Loamy sand	1.15	345.00
	2.45	Loamy sand	1.22	138.00
	3.05	Sand	1.35	518.00
Mohanpur	0	Loam	1.37	1.80
	0.15	Loam	1.64	0.60
	0.30	Loam	1.59	10.20
	0.90	Silt loam	1.64	1.80
	1.85	Sandy loam	1.42	33.00
	3.05	Sandy loam	1.46	51.60
Bagmara	0	Sandy loam	1.56	0.02
	0.15	Sandy loam	1.43	2.4
	0.30	Sandy loam	1.37	13.8
	0.60	Sandy loam	1.39	55.3
	1.25	Loam	1.41	10.4
	1.85	Loam	1.49	5.2
	2.45	Loam	1.6	2.1
	3.05	Clay loam	1.68	0.02

**Table 2:** Properties of aquifer sediments at Manda, Mohanpur and Bagmara

Location	Depth (m)	Aquifer	Effective grain size (mm)		Uniformity coefficient	Hydraulic conductivity (m/h)
			*D*_10_	*D*_60_		
Manda	12–15	1ST	0.17	0.21	1.23	7.30
	18–21	1ST	0.17	0.28	1.64	5.20
	27–30	2ND	0.12	0.24	1.96	3.60
	33–36	2ND	0.18	0.4	2.29	7.72
	36–39	2ND	0.19	0.6	3.16	11.70
Mohanpur	24–30	1ST	0.18	0.42	2.33	8.20
	36–39	2ND	0.16	0.27	1.69	6.40
	42–45	2ND	0.17	0.25	1.47	6.20
Bagmara	12–15	1ST	0.08	0.21	2.80	1.40
	15–18	1ST	0.16	0.23	1.44	6.45
	21–24	1ST	0.18	0.44	2.44	11.66
	27–30	1ST	0.22	0.42	1.90	15.70
	30–33	1ST	0.20	0.42	2.10	11.52
	33–36	1ST	0.18	0.40	2.22	10.50
	36–39	1ST	0.18	0.42	2.33	10.50

In Manda, during the wet season (May–August) in May, bacterial contamination (FC, FS or both) was observed in monitoring wells 1, 2, 5 and 6 which were installed at the lateral and vertical distances of 2–4.5 and 18–21 m, respectively, from the pit latrine (Table 3). An existing tubewell 18 m deep and located 9 m horizontally from the pit latrine did not show bacterial contamination. The results of the existing tubewell were consistent with the monitoring wells. Those monitoring wells were also contaminated during midterm (September–December) sampling. In the dry season, no contamination was found in monitoring wells 1 and 2 which were contaminated during wet and midterm (September–December) samplings. The monitoring well 7 was found to be contaminated during the midterm and dry season samplings. The monitoring wells 3, 4 and 8 were found free of contamination in all seasons. The monitoring wells were in the south east direction.

**Table 3:** Microbiological contamination of water samples of monitoring wells and nearest existing tubewell

Well no.	Depth of well (m)	Distance of pit (m)	FC/100 ml Wet season	FS/100 ml	FC/100 ml Midterm	FS/100 ml	FC/100 ml Dry season	FS/100 ml
Manda								
M-1	18.0	2.0	8	2	10	77	0	0
M-2	18.0	4.5	2	0	4	0	0	0
M-3	18.0	7.0	0	0	0	0	0	0
M-4	18.0	9.0	0	0	0	0	0	0
M-5	21.0	2.0	5	1	2	0	3	6
M-6	18.0	2.0	17	6	12	0	0	1
M-7	40.5	2.0	0	0	0	2	4	2
M-8	40.5	4.5	0	0	0	0	0	0
N	18.0	9.0	0	0	0	0	0	0
Mohanpur								
M-1	31.0	2.0	0	2	0	0	8	10
M-2	47.0	2.0	0	0	0	0	0	0
M-3	31.0	4.5	0	0	0	0	0	0
M-4	31.0	7.0	0	0	0	0	0	0
N	39.6	15.0	0	0	0	0	0	0
Bagmara								
M-1	27.5	2.0	2	68	2	2	1	0
M-2	27.5	2.0	4	3	1	0	0	0
M-3	27.5	2.0	1	2	0	1	1	1
M-4	42.5	2.0	0	0	1	60	11	0
M-5	42.5	4.5	0	0	0	0	0	0
M-6	27.5	4.5	0	0	0	0	0	0
M-7	27.5	7.0	0	0	0	0	0	0
M-8	27.5	9.0	0	0	0	0	0	0
N	27.5	8.5	0	0	0	0	0	0

*N* nearest existing tubewells, *M* monitoring well, *FC* faecal coliforms, *FS* faecal streptococci

### Mohanpur study site

In Mohanpur, the monitoring well logs showed that the upper or first aquifer was encountered between depths 23 and 33 m from the surface and was dominated by brown and grey fine sand (Fig. 4). The first aquifer was overlain by a grey and brown sticky clay layer which was 23 m thick. The lower or second aquifer was encountered below 37 m consisting of brown and grey fine sand mixing with medium and very fine sand and overlain by a 4-m-thick brown clay layer. The maximum and minimum levels of the groundwater table were found to be 14 and 4 m, respectively, below ground surface which were the same for both aquifers, indicating that they were hydraulically connected.

The soil texture of Mohanpur was composed of mainly loam and sandy loam (Table 1). The bulk density of soil ranged from 1.37 to 1.64 gm/cm^3^. The saturated hydraulic conductivity of soil was 0.60–51.60 mm/h. The calculated Uc of the aquifer sediments of Mohanpur was 2.33 for the first aquifer and 1.47–1.69 for the second aquifer. Hydraulic conductivities were 8.2 and 6.2–6.4 m/day for the first and the second aquifers, respectively.

At Mohanpur, during the wet and dry seasons, bacterial contamination was observed in monitoring well 1, which was 2 m away from the pit latrine at a depth of 31 m (Table 3). The existing tubewell which was 15 m away from the monitoring well was found to be FC and FS free during the study period. All other monitoring wells were also found free from contamination. The existing tubewell and the monitoring wells were located in the southeast direction from the latrine.

### Bagmara study site

In the monitoring well logs of Bagmara (Fig. 5), the first aquifer extended from 18 to 43 m below the surface consisting of grey fine and medium sand. The top 18-m clay layer may act as a barrier for surface contaminants.

The soil texture of Bagmara was composed of mainly loam and sandy loam (Table 1). The bulk density of soil was measured between 1.37 and 1.68 gm/cm^3^. The saturated hydraulic conductivity of soil was 0.02–55.30 mm/h. The estimated Uc of the aquifer sediments of Bagmara was 1.44–2.8, and hydraulic conductivities were calculated between 1.4 and 15.7 m/day in the first aquifer.

At Bagmara, bacterial contamination was observed in monitoring wells 1 and 3 in all seasons. Monitoring well 2 was contaminated in the wet season and midterm sampling but not in the dry season. The monitoring wells 1–3 had lateral and vertical distances of 2.0 and 27.5 m, respectively. Monitoring well 4 was found to be contaminated in the midterm sampling and dry season with lateral and vertical distances of 2.0 and 42.5 m, respectively. However, bacterial contamination was not observed in the monitoring wells 5–8 as well as existing tubewell having lateral and vertical distances of more than 4.5 and 27.5 m, respectively.

## Discussion

Results of the study suggested that the thickness of the first, i.e. surface clay, layer played an important role in protecting the aquifer from contamination of the nearby pit latrine. Amongst the three areas, the lowest contamination was found in Mohanpur, which had a 23-m-thick clay layer. This aquitard acted as a barrier for both vertical and horizontal movement of the bacteria. In Manda and Mohanpur, there was a second clay layer between the first and second aquifers which acted as a barrier for the second aquifer. However, Bagmara lacked this second clay layer. Therefore, the aquifer of Bagmara might be more vulnerable to contamination than Manda and Mohanpur. The highest contamination was observed in Manda, where the first clay layer was the thinnest amongst the three studied areas. The Uc of all the sediment samples were below 4 which indicated that the aquifer sediments in all locations were well sorted [26].

Monitoring wells in Manda having 18–21 m depths and 2–4.5 m away from the pit latrine were contaminated in the wet and midterm samplings, while in the dry season, monitoring wells 1 and 2 were free from contamination (Table 3). Again in the wet season in 12 May 2008, at greater depth, i.e. 40.5 m, the monitoring well was found free from contamination. Therefore, contamination varied according to the seasons as well as the lateral and vertical distances of the monitoring wells. Previous study [19] also supports more contamination of *E. coli* during the wet season (61 %) than the dry season (9 %) in shallow wells. Infiltration of faecal contamination into the shallow aquifer is most likely during the early monsoon under favourable hydraulic gradient [19] and shallow water table.

In Manda, the monitoring wells were established in two aquifers. The first and second aquifers had 9- and 3-m-thick silty clay layers, respectively. As contamination occurred in both aquifers, the two clay layers might not be thick enough to act as a barrier against bacterial movement. The characteristics of the soil in Manda indicated that microbial flow with water would be very rapid due to the cohesive nature and high hydraulic conductivity of the soil (Table 1). Microbe attachment is assumed to be either irreversible, where microbes are permanently filtered from the mobile liquid phase, or reversible, where microbes can reenter the flowing liquid [10]. Model implies that microbes are irreversibly attached to the solid phase, and the rate of attachment is related to the probability of a collision with the surface of the solid phase [29]. Therefore, the expected transport of bacteria might not occur always though the other hydrogeological conditions remained the same.

In the Mohanpur area, both the first and second aquifers were safe from surface contamination because of a sustainably thick sticky clay layer (23 m) at the surface. Moreover, a 4-m-thick sticky clay layer overlying the second aquifer might have additional protection of second aquifer against contamination. The characteristics of soil of Mohanpur depicted that microbial flow with water would be slow due to compactness and medium hydraulic conductivity of the soil. Results from existing wells showed no bacterial contamination of groundwater in that area.

In the Bagmara area, all the monitoring wells were installed in one aquifer at depths of 27.5 to 42.5 m. All the monitoring wells which were 2 m away from the pit latrine were found contaminated. Monitoring wells at lateral distances of more than 2 m were found free from bacterial contamination. The lateral distances in relation to hydrogeological conditions thus played an important role in determining contamination of the monitoring wells in Bagmara. The characteristics of soil of Bagmara indicated that microbial movement with water would be faster than Mohanpur as the first aquitard was not as compact as Mohanpur because it was composed of silt, fine and grey fine sands.

The results of the present study suggested that the contamination of groundwater from a pit latrine depended mainly on the lateral and vertical distances of the tubewells as well as the hydrogeological conditions of the particular area. A sustainably thick sticky clay layer, i.e. aquitard at the surface, was found to act as a good barrier for bacterial movement and prohibited the contamination of the aquifer. Simulations using a two-population model with parameters found in these experiments showed that bacterial concentrations would rapidly decrease within the first metre of transport but would decrease at a much slower rate over distances up to 10 m because of the low irreversible attachment rate of the second population. In these situations, long-distance transport of *E. coli* is determined mainly by decay rates [10].

These preliminary data indicated that no countrywide uniform guideline can be developed to install tubewells at a safe distance from nearby pit latrines in Bangladesh as the hydrogeological conditions vary from area to area.

## Conclusions

Pit latrines enhanced microbial contamination of adjacent shallow tubewell water where hydrogeological conditions (i.e. thickness and hydraulic properties such as hydraulic conductivity of surface clay aquitard, depth of groundwater table and groundwater flow direction) played important role on the transport of bacteria. Existence and level of contamination of bacteria differed in different hydrogeological conditions in both lateral and vertical distances, and where the surface clay was thick and compact, there was less or no contamination. Where there was a contamination, the level also varied at different seasons. During monsoon, the contamination was higher due to higher infiltration rate of precipitation water and shallow depth to water table. The present study did not produce sufficient data to develop general guidelines for the entire Bangladesh for the minimum safe distance of a tubewell from a pit latrine. Therefore, further studies need to be conducted including more physiographic divisions of Bangladesh with different hydrogeological conditions. Though microbiological contamination of the groundwater was found, most wells sampled showed good bacteriological quality of water, mostly where the hydrogeological conditions did not allow the transport of bacteria.

## Abbreviations

CFU: Colony-forming unit
DFID: Department of International Development
FC: Faecal coliforms
FS: Faecal streptococci
icddr,b: International Center for Diarrhoeal Disease Research, Bangladesh
mFC: Membrane faecal coliforms
SHARE: Sanitation and Hygiene Applied Research Equity
UC: Uniformity coefficient
VERC: Village Education Resource Center

## References

[1] UNICEF. Situation assessment and analysis of children and women in Bangladesh. Dhaka: UNICEF; 2009.

[2] Wu J, Yunus M, Streatfield PK, van Geen A, Escamilla V, Akita Y, Serre M, Emch M (2011). Impact of tubewell access and tubewell depth on childhood diarrhea in Matlab, Bangladesh. Environ. Health.

[3] Macdonald D, Ahmed KM, Islam MS, Lawrence A, Khandker ZZ (1999). Pit latrines—a source of contamination in peri-urban Dhaka?. Waterlines.

[4] Rahman SH, Ahmed S, Zakariya M (2009). Investigation of shallow tube-well water quality considering the influence of nearby latrines in a rural village of Bangladesh. Trends and sustainability of groundwater in highly stressed aquifers. Proceeding of Symposium JS.2at the Joint IAHS and IAH Convention.

[5] NIPORT (2005). Bangladesh demographic and health survey.

[6] Islam MS, Siddika A, Khan MNH, Goldar MM, Sadique MA, Kabir ANMH, Huq A, Colwell RR (2001). Microbiological analysis of tube-well water in a rural area of Bangladesh. Appl Environ Microbiol.

[7] Luby S, Islam MS, Johnston R (2006). Chlorine spot treatment of flooded tube wells, an efficacy trial. J Appl Microbiol.

[8] Luby SP, Gupta SK, Sheikh MA, Johnston RB, Ram PK, Islam MS (2008). Tubewell water quality and predictors of contamination in three flood-prone areas in Bangladesh. J App Microbiol.

[9] Lewis WJ, Foster SS, Drasar BS (1982). The risk of groundwater pollution by on-site sanitation in developing countries.

[10] Foppen JWA, and Schijven JF. Evaluation of data from the literature on the transport and survival of Escherichia coli and thermotolerant coliforms in aquifers under saturated conditions. Water Res. 2006;40:401–26. 10.1016/j.watres.2005.11.018 16434075

[11] Statistical yearbook of Bangladesh 2007. Dhaka: Bangladesh Bureau of Statistics, Planning Division, Ministry of Planning, Government of the People’s Republic of Bangladesh; 2008. p. 515.

[12] Rahman SH, Fakhruddin ANM, Uddin MJ, Zaman MS, Talukder A, Adyel TM, Sarker MR (2013). Water quality of shallow tubewells as affected by sanitary latrines and groundwater flow. J Bang Acad of Sci.

[13] Feighery J, Mailloux BJ, Ferguson AS, Ahmed KM, van Geen A, Culligan PJ (2013). Transport of E. coli in aquifer sediments of Bangladesh: implications for widespread microbial contamination of groundwater. Water Resour. Res.

[14] McArthur JM, Sikdar PK, Nath B, Grassineau N, Marshall JD, Banerjee DM (2012). Sedimentological control on Mn, and other trace elements, in groundwater of the Bengal Delta. Environ Sci Technol.

[15] Tufenkji N (2007). Modeling microbial transport in porous media: traditional approaches and recent developments. Adv Water Resour.

[16] Barrett MH, Howard AG, Howard KWF, Israfilov RG (2002). Urban groundwater and sanitation—developed and developing countries. Current problems of hydrogeology in urban areas.

[17] Nataraju C (2001). Studies on vulnerability of groundwater to pollution, its potential and quantification methodologies (Bangalore rural and urban districts, Karnataka, India).

[18] Lawrence AR, Macdonald DMJ, Howard AG, Barrett MH, Pedley S, Ahmed KM (2001). Guidelines for assessing the risk to groundwater from on-site sanitation.

[19] Knappett PSK, McKay LD, Layton A, Williams DE, Alam MJ, Huq MR, Mey J, Feighery JE, Culligan PJ, Mailloux BJ, Zhuang J, Escamilla V, Emch M, Perfect E, Sayler GS, Ahmed KM, Van Geen A (2012). Implications of fecal bacteria input from latrine-polluted ponds for wells in sandy aquifers. Environ Sci Technol.

[20] Leber J, Rahman MM, Ahmed KM, Mailloux B, Van Geen A (2011). Contrasting influence of geology on *E. coli* and arsenic in aquifers of Bangladesh. Ground Water.

[21] Alam MK, Hasan AKM, Khan MR, Whitney JW (1990). Geological map of Bangladesh.

[22] Land Resources Appraisal of Bangladesh for Agricultural Development Report No. 2. Rome: United Nations Development Programme and Food and Agricultural Organization; 1988: p. 1–570.

[23] O'neal AM (1952). A key for evaluating soil permeability by means of certain field clues. Soil Sci Soc Am J.

[24] Bouyoucos GJ (1962). Hydrometer method improved for making particle size analyses of soils. Agron J.

[25] Klute A, Klute A (1986). Water retention: laboratory methods. Methods of soil analysis: part 1—physical and mineralogical methods.

[26] Hazen A (1982). Some physical properties of sands and gravels with special reference to their use in filtration.

[27] World Health Organization. Guidelines for drinking-water quality: recommendations (Vol. 1). World Health Organization; 2004.

[28] Islam MS, Brooks A, Kabir MS, Jahid IK, Islam MS, Goswami D (2007). Faecal contamination of drinking water sources of Dhaka city during the 2004 flood in Bangladesh and use of disinfectants for water treatment. J Appl Microbiol.

[29] Happel J (1958). Viscous flow in multiparticle systems: slow motion of fluids relative to beds of spherical particles. AIChE J.

